# Isolated left bronchial isomerism that was incidentally detected as a severe obstructive ventilatory disturbance in an asymptomatic adult patient: A case report

**DOI:** 10.1097/MD.0000000000020246

**Published:** 2020-05-29

**Authors:** Manbong Heo, Jong Hwan Jeong, Jung Wan You, Ju-Young Kim, Mi Jung Park, Kyung Nyeo Jeon, Jong Deog Lee, Seung Jun Lee

**Affiliations:** a Department of Internal Medicine; b Department of Radiology, Gyeongsang National University Hospital; c Department of Radiology, Gyeongsang National University Changwon Hospital, Changwon, Gyeongsang National University School of Medicine, Jinju, Republic of Korea.

**Keywords:** chronic obstructive pulmonary disease, left bronchial isomerism, lung function, pulmonary function test, young adult

## Abstract

**Rationale:**

Left bronchial isomerism is generally associated with abnormal arrangement of the atrium and abdominal viscera; therefore, its diagnosis is confirmed in early childhood.

**Patient concerns:**

Here we report a rare case involving a 36-year-old man with isolated left bronchial isomerism that presented as an asymptomatic severe obstructive ventilatory disturbance during pulmonary function tests performed as part of routine assessments for an orbital wall fracture. The patient was a current smoker and did not show any respiratory symptoms.

**Diagnosis:**

Chest computed tomography revealed left bronchial isomerism, and further tests showed that there was no involvement of other organs.

**Interventions:**

We recommended smoking cessation and the long-term use of an inhaled long-acting bronchodilator.

**Outcomes:**

The findings from this case highlight the causative role of left bronchial isomerism in asymptomatic adults with chronic obstructive pulmonary disease.

**Lessons:**

Physicians should consider this condition as a cause of obstructive ventilatory disturbances in asymptomatic adult patients.

## 1 Introduction

Isomerism is defined as the absence of normal right- and left-sided internal organs such as the lungs, heart, liver, and gut.^[1]^ Left bronchial isomerism is characterized by the presence of a morphologically identical left bronchus and lung on the right side, and it is generally associated with congenital heart disease characterized by an identical left atrium on the right side of the heart.^[2]^ Left bronchial isomerism can also be associated with polysplenia, a midline liver, and gut malrotation.^[3]^ Thus, patients with left bronchial isomerism associated with congenital heterotaxy syndrome are diagnosed at the beginning of life and show a poor prognosis. Here we report a rare case of isolated left bronchial isomerism that presented as an asymptomatic severe obstructive ventilatory disturbance in routine pulmonary function tests for a young adult. Patient has provided informed consent for publication of the case.

## 2 Case report

**Figure 1:**
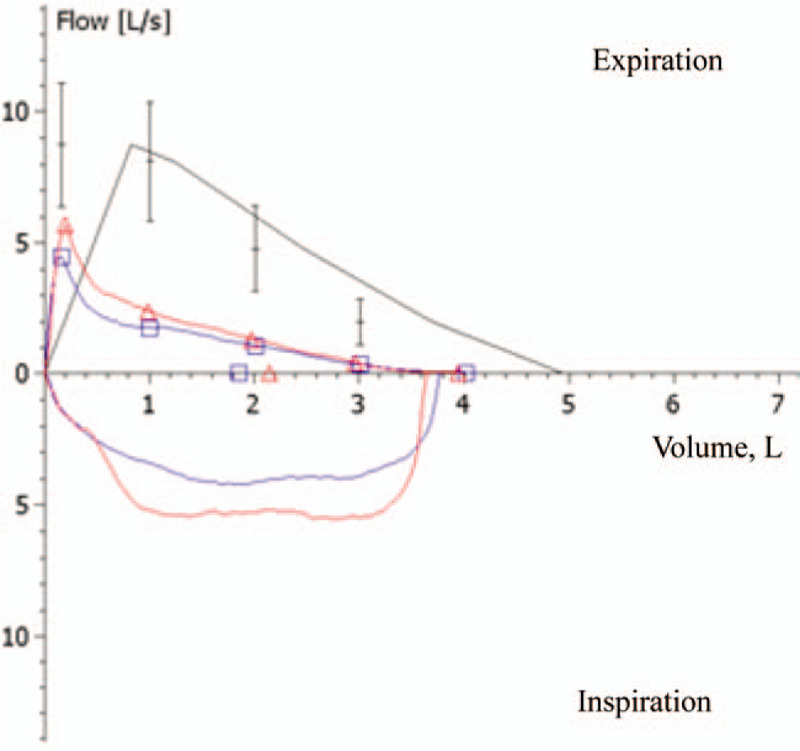
Flow/volume curves before (blue color) and after (red color) inhalation of a short-acting bronchodilator for an asymptomatic, 36-yr-old man with incidentally detected isolated left bronchial isomerism.

A 36-year-old man was referred to our pulmonary clinic for further evaluation of abnormal pulmonary function test findings during preoperative evaluation of an orbital wall fracture. His forced expiratory volume in 1 second (FEV_1_), forced vital capacity, and FEV_1_/forced vital capacity ratio were 1.84 L (45.8%), 4.01 L (81.3%), and 46.0%, respectively (Fig. 1). He was a current smoker with a smoking history of 30 pack-years. His height and body weight were 170 cm and 82 kg, respectively, and his medical history was unremarkable.

On examination, he appeared healthy, alert, and oriented. His vital signs were as follows: blood pressure, 125/75 mm Hg; body temperature, 36.6°C; heart rate, 72 beats/min; respiratory rate, 18 breaths/min; and oxygen saturation, 99% on room air. His breath sounds were normal, with no wheezing and crackles.

**Figure 2:**
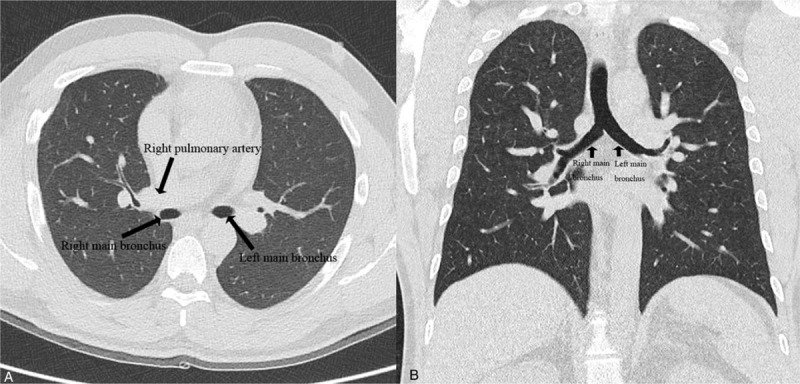
Axial (panel A) and coronal (panel B) low-dose computed tomography images for an asymptomatic, 36-yr-old man with incidentally detected isolated left bronchial isomerism. The images show a hyparterial right main bronchus with bilateral bilobed lungs.

**Figure 3:**
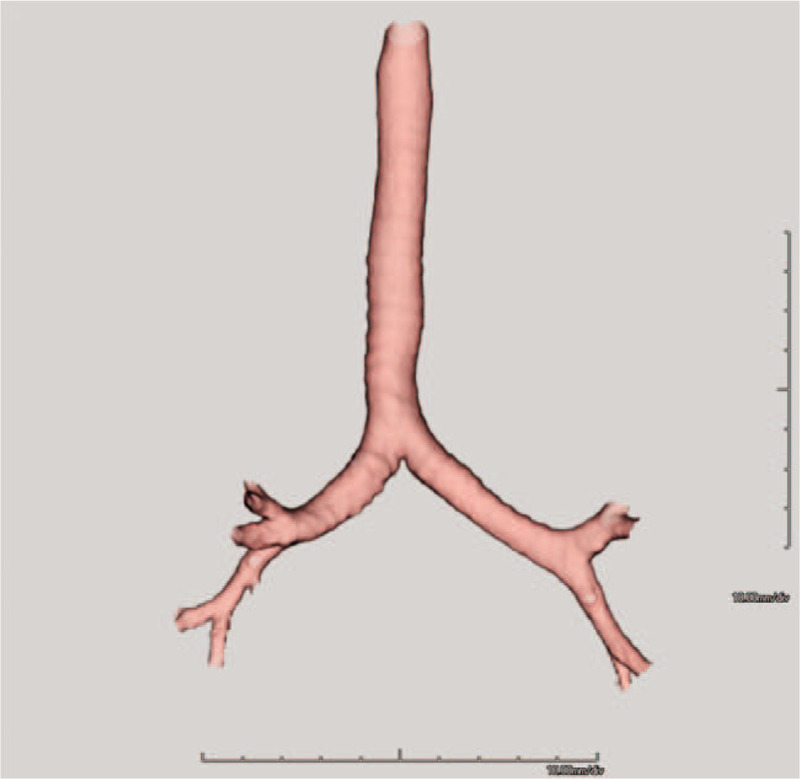
A 3-dimensional reconstructed computed tomography image for an asymptomatic, 36-yr-old man with incidentally detected isolated left bronchial isomerism. The image shows identical, long left main bronchi on the right and left sides and the absence of a right upper lobe bronchus.

Low-dose chest computed tomography (CT) was performed to screen for lung cancer as per the patient's request. Axial (Fig. 2A) and coronal (Fig. 2B) images showed a bilateral bilobed lungs with hyparterial right main bronchus, findings indicative of left bronchial isomerism. A 3-dimensional reconstructed CT image showed identical, long left main bronchi on the right and left sides and the absence of a right upper lobe bronchus (Fig. 3). Pulmonary emphysema was not seen. Echocardiography and abdominal ultrasound did not reveal any structural abnormalities in the internal organs. Moreover, the patient had no subjective respiratory symptoms despite the severely declined lung function. A bronchodilator test was performed and the diffusing capacity of the lungs for carbon monoxide (DLco), total lung capacity (TLC), and residual volume (RV) were measured. His FEV_1_ increased to 300 mL after salbutamol inhalation, showing a change of 16.2% in the percentage predicted value (Fig. 1). The percentage predicted values for DLco, TLC, and RV were 112%, 95%, and 103%, respectively. The distance covered in the 6-minute walk test was 460 m. On the basis of all these findings, he was diagnosed with isolated left bronchial isomerism and recommended smoking cessation along with long-term use of an inhaled long-acting bronchodilator.

## 3 Discussion

In this report, we described a case of left bronchial isomerism without involvement of other organs in an asymptmatic adult patient. Severe airway obstruction was incidentally detected, although the lung volumes and diffusion capacity were normal. Despite the severe decline in his lung function, there were no subjective symptoms.

Bronchial isomerism is usually a component of congenital heterotaxy syndrome that is diagnosed in infancy or early childhood.^[4]^ Left bronchial isomerism without heterotaxy diagnosed in asymptomatic adults is an extremely rare phenomenon, and only a few cases have been reported till date.^[5,6]^ Huang et al. and Read et al. reported cases of left bronchial isomerism that was discovered during lung cancer surgery^[6,7]^ and described and showed the intraoperative bronchial anatomy of left bronchial isomerism in detail. Suen et al reported left bronchial isomerism that was incidentally found during the diagnostic process of abnormal chest radiograph findings in a 42-years-old, asymptomatic woman. The patient presented with an incidental chest radiograph finding of a right lung nodule that turned out to be benign, and her left bronchial isomerism was confirmed via bronchoscopy.^[5]^ We recommended bronchoscopy for the definitive diagnosis of left bronchial isomerism to our patient; however, he refused to undergo the same. Nevertheless, careful review of chest CT images and 3-dimensional CT image reconstruction were adequate to confirm the presence of left bronchial isomerism in this case.

The most interesting findings in the present case were the pulmonary function test results. Although spirometry indicated a severe obstructive ventilatory disturbance, DL_CO_ and lung volumes, including TLC and RV, were normal. Furthermore, there were no signs indicating chronic obstructive pulmonary disease (COPD), such as emphysema and air-trapping, on his chest CT images. In addition, the patient did not exhibit any symptoms of COPD, such as chronic cough, sputum, and dyspnea on exertion. It remains uncertain whether an obstructive ventilatory disturbance is a usual finding in patients with left bronchial isomerism. Spirometry was not performed for the case reported by Suen et al,^[5]^ while pulmonary function tests were performed for 2 other cases of left bronchial isomerism reported by Lee et al and Bush et al.^[1,2]^ Although these 2 cases involved teenage patients with persistent coughing and wheezing, the lung function parameters were very similar to those for the present case. However, both patients were symptomatic and had a history of respiratory diseases,^[1,2]^ whereas our patient was asymptomatic and provided negative answers when asked about any history of respiratory diseases during his childhood and juvenile years. Meanwhile, he exhibited a positive response in the bronchodilator test, which was not performed for the 2 teenage patients. Instead, the authors had identified bronchomalacia during fiberoptic bronchoscopy.^[1,2]^ These findings collectively suggest that patients with left bronchial isomerism generally show marked airway obstruction with a normal diffusion capacity and lung volume and a positive bronchodilator response.

With regard to the optimal treatment of choice for left bronchial isomerism, no reports have been published till date. We speculate that long-acting bronchodilators and inhaled corticosteroids could be good candidate drugs, considering the findings of spirometry and bronchodilator tests. A long-acting β-agonist and inhaled corticosteroid were prescribed to the patient reported by Bush et al, although they were ineffective. Although the bronchodilator response was positive in our case, the patient did not have any symptoms of asthma during his lifetime. We prescribed tiotropium in consideration of his smoking history and spirometry results.

In conclusion, the findings from this case highlight the causative role of left bronchial isomerism in asymptomatic adults with COPD. Physicians should consider this condition as a cause of obstructive ventilatory disturbances in asymptomatic adult patients.

## Author contributions

**Conceptualization:** Jong Hwan Jeong, Ju-Young Kim, Mi Jung Park, Jong Deog Lee, Seung Jun Lee.

**Data curation:** Jong Hwan Jeong, Ju-Young Kim, Mi Jung Park, Jong Deog Lee, Seung Jun Lee.

**Investigation:** Manbong Heo.

**Methodology:** Manbong Heo.

**Project administration:** Jong Hwan Jeong, Ju-Young Kim.

**Software:** Mi Jung Park, Kyung Nyeo Jeon.

**Supervision:** Kyung Nyeo Jeon.

**Visualization:** Mi Jung Park, Kyung Nyeo Jeon.

**Writing – original draft:** Manbong Heo.

**Writing – review & editing:** Jong Deog Lee, Seung Jun Lee.
